# Endothelial progenitor cells and cerebral small vessel disease in *APOE4* carriers

**DOI:** 10.1016/j.cccb.2025.100378

**Published:** 2025-02-11

**Authors:** Arunima Kapoor, Shubir Dutt, Amy Nguyen, Trevor Lohman, Aimée Gaubert, John Paul M. Alitin, Isabel J Sible, Anisa Marshall, Fatemah Shenasa, Allison C Engstrom, David Robert Bradford, Kathleen Rodgers, Daniel A Nation

**Affiliations:** a Department of Psychological Science, University of California, Irvine, Irvine, CA, USA; b Department of Psychology, University of Southern California, Los Angeles, CA, USA; c Leonard Davis School of Gerontology, University of Southern California, Los Angeles, CA, USA;; d Center for Innovations in Brain Science, Department of Pharmacology, University of Arizona, Tucson, AZ, USA;; e Department of Physiology and Neuroscience, Zilkha Neurogenetic Institute, Keck School of Medicine, Los Angeles, CA, USA

**Keywords:** Endothelial progenitor cells, White matter hyperintensities, Small vessel disease, APOE4

## Abstract

*APOE4* carriers at genetic risk for Alzheimer's disease exhibit early cerebrovascular dysfunction, which may be triggered by endothelial dysfunction. Endothelial progenitor cells (EPCs) represent cell populations involved in promoting angiogenesis and facilitating vascular repair in response to injury. We examined whether elevated EPCs are associated with lower cerebral small vessel disease burden in *APOE4* carriers prior to cognitive decline. Independently living older adults (*N* = 109, mean age = 70.5 years; SD = 7.9; 34.9 % male) free of dementia or clinical stroke underwent brain MRI and venipuncture. Small vessel disease was determined using a validated scale. White matter hyperintensity (WMH) volume was determined using the lesion segmentation toolbox. PBMCs were cultured and EPCs were defined as number of colony forming units in vitro. Regression analysis revealed an association between average number of EPC colonies and lower small vessel disease load (*p* = .026) and WMH volume (*p* = .002), in *APOE4* carriers. Findings suggest that EPC colony count may indicate activation of mechanisms which protect cerebrovascular function in *APOE4* carriers prior to the development of cognitive decline.

## Highlights

• *APOE4* carriers exhibit cerebrovascular dysfunction.
• Endothelial progenitor cells (EPC) represent cell populations involved in facilitating vascular repair.
• In *APOE4* carriers, EPC colony count is associated with cerebral small vessel disease.
• EPC colony count may indicate activation of mechanisms which protect cerebrovascular function in *APOE4* carriers prior to the development of cognitive decline.

## Endothelial progenitor cells and cerebrovascular function in *APOE4* carriers

The ε4 allele of the apolipoprotein E gene (*APOE4*) confers the strongest genetic risk for developing late-onset Alzheimer's disease [1,2]. *APOE4* is linked to increased cerebral amyloid beta retention, changes in lipid metabolism and inflammation [[3], [4], [5]]. Recently, *APOE4* has also been associated with cerebrovascular dysfunction and may increase risk of Alzheimer's disease via a vascular mechanism [[6], [7], [8]]. It has been hypothesized that *APOE4* may influence pericyte degeneration, thinning of the microvascular basement membrane, endothelial dysfunction and breakdown of the blood-brain barrier [7,[9], [10], [11], [12], [13]]. These processes may result in changes in cerebral microvasculature in *APOE4* carriers [14,15].

Damage to the endothelium can influence transport of nutrients and waste, the ability of the vessel to contract and dilate, and may impact vascular pulsatility [16]. Endothelial progenitor cells (EPCs) represent a heterogeneous cell population that facilitate angiogenesis and vasculogenesis and mobilize in response to vascular injury to promote vascular repair [17]. Prior studies have reported mobilization of these cells in response to a range of vascular conditions, including arteriovenous malformations [18], cerebral autosomal dominant arteriopathy with subcortical infarcts and leukoencephalopathy (CADASIL) [19], ischemic stroke [20], hemorrhage [21] and moyamoya disease [22]. Across these conditions, mobilization of EPCs is hypothesized to promote vascular remodeling and repair dysfunctional endothelium [23,24]. Transplantation of EPCs has been proposed as potential treatment for vascular damage [25] and animal models have demonstrated that EPCs may be able to protect blood-brain barrier integrity after focal ischemia [26]. Levels of EPCs may also be altered in individuals with Alzheimer's disease and could indicate impaired hematopoietic and endothelial function [[27], [28], [29], [30]].

Moreover, mobilization of EPCs may play a role in cerebrovascular dysfunction in those at genetic risk of Alzheimer's disease. While prior studies have observed an association between EPCs and both vascular risk factors and cerebral small vessel disease [31,32], few have examined EPCs related to cerebrovascular dysfunction in *APOE4* carriers. In the current study, we examined whether EPC colony count is associated with burden of cerebral small vessel disease in *APOE4* carriers prior to development of cognitive decline. We hypothesized that greater EPC colony count will be associated with lower burden of cerebral small vessel disease, particularly in *APOE4* carriers.

## Methods

### Participants

Participants were recruited from the community and all study procedures were conducted at the Vascular Senescence and Cognition (VaSC) Lab at the University of Southern California (USC) and University of California, Irvine (UCI). Older adults aged 55 to 90 years who were living independently were included. Exclusion criteria included no history of clinical stroke, dementia, major psychiatric disorder, MRI contraindication, current organ failure, chronic kidney disease and other systemic or neurological illness(s) or medication(s) that may impact central nervous system function, including monogenic forms of small vessel disease. History of vascular risk factors, including hypertension, dyslipidemia, diabetes, as well as general health history and family history of dementia, was determined by clinical interview. This study was approved by the University of Southern California and University of California, Irvine Institutional Review Boards and conducted in accordance with the Declaration of Helsinki. All participants gave informed consent and underwent detailed clinical assessment to determine demographics and medical history, blood draw for quantification of EPC colony count and brain magnetic resonance imaging (MRI) for quantification of cerebral small vessel disease. White matter hyperintensity lesion segmentation and small vessel disease scoring were conducted by a rater who was blinded to EPC data at the time of data collection and processing.

### Endothelial progenitor cell (EPC) quantification

Venipuncture was performed after an overnight fast. EPC colonies was quantified using fresh blood samples in an in vitro assay. Briefly, peripheral blood mononuclear cells (PBMCs) were isolated by density gradient centrifugation and washed twice with DPBS + 2 % FBS at 120 x g for 10 min and 300 x g for 8 min at room temperature. Isolated PBMCs were washed, resuspended in EGM-2 BulletKit (Lonza), and seeded on type I collagen-coated 24-well plates at 1 × 10^6^ cells/well. PBMCs were incubated in a 5 % CO_2_ incubator at 37 °C. Early EPC colonies were defined as cluster with a central core of round cells surrounded by radiating spindle-shaped endothelial-like cells and manually counted on day 7 of culture [30]. Mean colony number across wells was calculated and log-transformed values were used in the analysis.

### *APOE* genotyping

Apolipoprotein (*APOE*) genotyping was conducted on the blood cell pellet fraction obtained from plasma separation. DNA was isolated from the pellet fraction using the PureLink Genomic DNA Mini Kit (Thermo Fisher). Genotyping was conducted on isolated DNA using the TaqMan SNP Genotyping Assay (Thermo Fisher) on an Applied Biosystems 7300 Real Time PCR System. *APOE* gene SNPs were assessed for dbSNP IDs rs429358 and rs7412. Allelic discrimination was conducted using the included qPCR software. The *APOE*-ε4 allele was designated as rs429358-C + rs7412-C.

### Neuroimaging

All participants underwent brain MRI on a 3T scanner (Siemens MAGNETOM Prisma System). The following sequences were examined for the current analysis: 3D T1-weighted MPRAGE anatomical scan for qualitative assessment of brain structures and abnormalities (scan parameters: TR = 2300 ms; TE = 2.98 ms; TI = 900 ms; flip angle = 9 deg; FOV = 256 mm; resolution = 1.0 × 1.0 × 1.2 mm3; Scan time = 9 mins), FLAIR for evaluation and segmentation of white matter hyperintensities and differentiation of lacunes and perivascular spaces (scan parameters: TR = 10,000 ms; TE = 91.0 ms; flip angle = 150 deg; FOV = 220 mm; resolution = 0. 0.9 × 0.9 × 5.0 mm3; echo spacing = 8.31 ms; echo trains per slice = 8; scan time = 4 mins), T2-weighted scan for identification of enlarged perivascular spaces (scan parameters: TR = 10,000 ms; TE = 88 ms; flip angle = 120 deg; FOV = 210 mm; resolution = 0.8 × 0.8 × 3.5 mm3; echo spacing = 9.8 ms; echo trains per slice = 11; scan time = 2 mins), and T2∗-weighted imaging for identification of cerebral microbleeds (scan parameters: TR = 650 ms; TE = 20 ms; flip angle = 20 deg; FOV = 200 mm; resolution = 0.8 × 0.8 × 2.0 mm3; scan time = 6 mins).

#### Cerebral small vessel disease burden estimation

MRI markers were identified in accordance with established neuroimaging standards for cerebral small vessel disease [33] and scored by a blinded doctoral candidate (AK) trained by a board-certified neuroradiologist. To determine total MRI cerebral small vessel disease burden, all imaging markers were combined using a total cerebral small vessel disease score developed by Staals et al. (2014), which ranges from 0 to 4 and includes presence of white matter hyperintensities (1 point for Fazekas score 2–3), lacunes (1 point for ≥ 1), microbleeds (1 point for ≥ 1) and perivascular spaces (1 point for moderate to severe basal ganglia perivascular spaces).

#### White matter hyperintensity lesion segmentation

White matter lesions were segmented with the lesion growth algorithm implemented in the LST toolbox version 3.0.0 (www.statistical-modelling.de/lst.html) for SPM12 (Wellcome

Department of Cognitive Neurology, London, UK [34]). Initial threshold was set at 0.2 and visual inspection was conducted to determine optimal threshold for each individual; manual quality control check ensured no gross over- or under-estimation (i.e., each image was visually examined to ensure optimal segmentation).

### Statistical analyses

All analyses were performed using R Version 3.6.1, Prism Version 10.1.2, and IBM SPSS Statistics 29. Demographic variables were computed to characterize the sample. The relationship between average EPC colony count (independent predictor) and small vessel disease score, as well as white matter hyperintensities was examined using multiple linear regression. All analyses were adjusted for age and sex. Models predicting white matter hyperintensities also adjusted for total intracranial volume (ICV). Multicollinearity was assessed, with a variance inflation factor above 4 indicating significant multicollinearity. Average EPC colony count and white matter hyperintensity volume values were log-transformed to normalize the distribution prior to statistical analyses. The same analyses were conducted stratified by *APOE4* carrier status. The slopes of the univariable linear regression examining the association between average EPC colony count (independent predictor) and small vessel disease score as well as white matter hyperintensities (dependent outcomes) for *APOE4* carriers and non-carriers were compared using the approach described by Zar (1984) in GraphPad Prism [35]. Significance threshold was set at *p* < .05. To adjust for multiple comparisons, we conducted a False Discovery Rate (FDR) correction using the Benjamini-Hochberg method, with an adjusted *p* < 0.05 threshold. Adjusted p-values based on these procedures are included in the results.

## Results

A total of 109 participants completed all procedures and were included in the current analysis. Age of study participants ranged from 55 to 90 years and years of education ranged from 6 to 20. Participant characteristics and vascular risk factors are reported in Table 1. Average EPC colony count (colonies per well) ranged from 0.50 to 61.7. Figures of these EPC colonies have been published previously [29]. The number of wells examined for each participant ranged from 1 to 18 (M = 8.59). The average within-individual variability in terms of standard deviations was 3.60 (i.e., average standard deviation between wells). Thirty-five (33.3 %) of participants were *APOE4* carriers. *APOE2/4* carriers were excluded from this analysis, given that *APOE2* is protective while *APOE4* increases risk. No differences in demographic variables were observed between *APOE4* carriers and non-carriers (Table 1). Cerebral small vessel disease score was correlated with white matter hyperintensity volume (*r* = 0.65, *n* = 78, *p* < .001).

**Table 1:** Participant characteristics, demographics and vascular risk factors.

	All (*N* = 109)	*APOE4* Carriers (*N* = 35)	*APOE4* Non-Carriers (*N* = 70)	p-value
Age (Years), M (SD)	70.5 (7.9)	68.7 (7.2)	71.1 (8.2)	.134
Sex Male, n (%)	38 (34.9)	15 (42.9)	22 (31.4)	.248
Education (Years), M (SD) *APOE4* Carrier (3/4 or 4/4, n (%)	16.4 (2.4) 35 (32.1)	16.3 (2.3)	16.5 (2.4)	.621
Hypertension, n (%)	39 (35.8)	12 (34.3)	26 (37.1)	.774
Dyslipidemia, n (%)	56 (52.3)	18 (52.9)	35 (50.7)	.832
Diabetes, n (%)	12 (11.0)	3 (8.6)	9 (12.9)	.515
Smoking History, n (%)	43 (39.4)	11 (31.4)	28 (40.0)	.392
TIA, n (%)	4 (3.7)	2 (5.7)	2 (8.6)	.471
Cardiovascular Disease, n (%)	8 (7.4)	1 (2.9)	7 (10.1)	.188
Atrial Fibrillation, n (%)	4 (3.7)	3 (8.6)	1 (1.4)	.074
Race, n (%) White Black Asian Other	78 (71.6) 10 (9.2) 15 (13.8) 6 (5.5)	28 (80.0) 2 (5.7) 5 (14.3) 0 (0.0)	47 (67.1) 7 (10.0) 10 (14.3) 6 (8.6)	.257
EPC Colonies (Colonies/Well), M (SD)	11.4 (12.4)	10.1 (10.3)	12.1 (13.5)	.442
Small Vessel Disease (Score), M (SD)	0.9 (0.9)	0.9 (1.0)	0.9 (0.9)	.811
Microbleeds (Present), n (%) Fazekas (Score 2–3), n (%) Lacunes (Present), n (%) Perivascular Spaces (Score 2–4), n (%)	11 (11.7) 39 (36.8) 17 (16.0) 30 (32.3)	5 (16.1) 15 (42.9) 5 (14.3) 7 (21.2)	6 (10.2) 21 (31.3) 11 (16.4) 20 (35.7)	
White Matter Hyperintensity Volume (mL), M (SD)	5.2 (5.9)	5.0 (5.1)	5.2 (6.3)	.850

*APOE4 carrier status was available for 105 participants; small vessel disease score was available for 82 participants; white matter hyperintensity volume was available for 100 participants; history of dyslipidemia was unknown for 2 participants (1 *APOE4* carrier, 1 non-carrier) and history of cardiovascular disease and atrial fibrillation was unknown for 1 participant (1 *APOE4* non-carrier); microbleeds data was missing for 15 participants (4 *APOE4* carriers, 11 non-carriers); Fazekas score and lacunes data was missing for 3 participants (3 *APOE4* non-carriers); perivascular spaces score was missing for 16 participants (2 *APOE4* carriers, 14 non-carriers).

*Note*: TIA = Transient Ischemic Attack; EPC = Endothelial Progenitor Cells; M = Mean; SD = Standard Deviation.

### EPC colony count and small vessel disease score

**Fig. 1:**
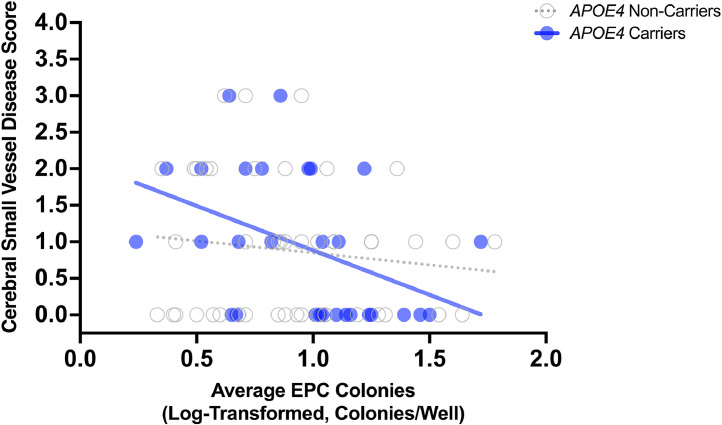
**Scatterplot of association between EPC colony count and small vessel disease burden in APOE4 carriers and non-carriers**. In *APOE4* carriers, higher EPC colony count was associated with lower small vessel disease burden. EPC colony count was not associated with small vessel disease burden in *APOE4* non-carriers. EPC colony count values were log-transformed.

In multiple regression analyses, EPC colony count was not associated with small vessel disease score after adjusting for age and sex (*B* = −0.48, 95 % CI [−1.02, 0.07], *p* = .085; *N* = 82; Table 2). When *APOE4* was added to this model, EPC colony count was marginally significantly associated with small vessel disease score (*B* = −0.55, 95 % CI [−1.13, 0.02], *p* = .059). When the *APOE4* x EPC colony count interaction term was added into the model, EPC colony count, *APOE4* and the *APOE4* x EPC colony count interaction was not associated with small vessel disease score. When stratified by *APOE4* carrier status, there was a stronger association between EPC colony count and small vessel disease in *APOE4* carriers (*B* = −1.35, 95 % CI [−2.53, −0.18], *p* = .026, adjusted *p* = .051; *N* = 29; Supplemental Table I) than not non-carriers (*B* = −0.30, 95 % CI [−0.99, 0.40], *p* = .398, adjusted *p* = .398; *N* = 49; Supplemental Table II; Fig. 1); however, the association in *APOE4* carriers did not survive FDR correction (adjusted *p* = .051) . In univariable regression, the difference between the slopes for *APOE4* carriers versus non-carriers was not significant (F(1, 74) = 2.04, *p* = .158; Fig. 1).

**Table 2:** Association between EPC colony count and small vessel disease burden adjusting for age and sex.

Variable	Unstandardized Coefficients		t	Sig.	95 % Confidence Interval for B	
	B	Std. Error			Lower Bound	Upper Bound
EPC Colony Count (Colonies/Well)*	−0.48	0.27	−1.74	0.085	−1.02	0.07
Age (Years)	0.04	0.01	3.06	0.003	0.01	0.07
Sex (Male)	0.26	0.21	1.23	0.224	−0.16	0.68

Dependent Variable: Small Vessel Disease Score*Values were log-transformed.

We further conducted a sensitivity analysis by recalculating the cerebral small vessel disease score by removing the points for white matter hyperintensities (i.e., 1 point for Fazekas score 2–3). This analysis yielded similar results; EPC colony count was associated with this modified small vessel disease score after adjusting for age and sex (*B* = −0.46, 95 % CI [−0.87, −0.05], *p* = .028). Moreover, when stratified by APOE4 carrier status, the association between EPC colony count and modified small vessel disease score was significant in APOE4 carriers (*B* = −0.77, 95 % CI [−1.53, −0.01], *p* = .047) but not non-carriers (*B* = −0.39, 95 % CI [−0.97, 0.20], *p* = .191]).

### EPC colony count and white matter hyperintensity volume

**Fig. 2:**
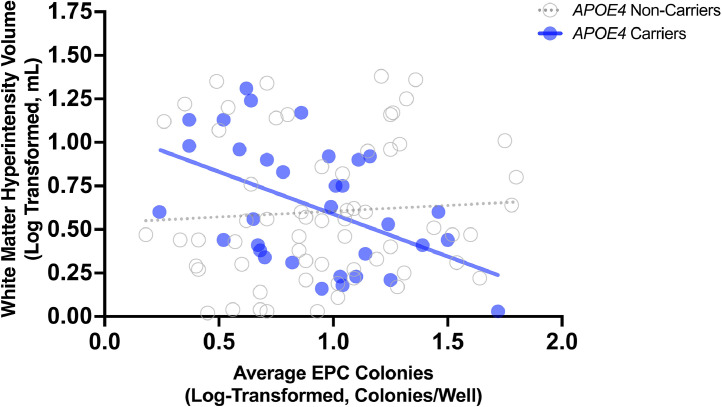
**Scatterplot of association between EPC colony count and white matter hyperintensity volume in APOE4 carriers and non-carriers.** In *APOE4* carriers, higher EPC colony count was associated with lower white matter hyperintensity volume. EPC colony count was not associated with white matter hyperintensity volume in *APOE4* non-carriers. EPC colony count and white matter hyperintensity volumes values were log-transformed.

Similarly, in multiple regression analyses, EPC colony count was not associated with white matter hyperintensity volume after adjusting for intracranial volume, age and sex (*B* = −0.06, 95 % CI [−0.23, 0.11], *p* = .499; *N* = 99; Table 3). When *APOE4* was added to this model, EPC colony count (*B* = −0.06, 95 % CI [−0.23, 0.12], *p* = .508) was not associated with white matter hyperintensity volume. When the *APOE4* x EPC colony count interaction term was added into the model, EPC colony count was not associated with white matter hyperintensity volume; however, *APOE4* status (*B* = 0.51, 95 % CI [0.14, 0.89], *p* = .008) and the *APOE4* x EPC colony count interaction (*B* = −0.45, 95 % CI [−0.83, −0.07], *p* = .020) were associated with white matter hyperintensity volume. When stratified by *APOE4* carrier status, the association between EPC colony count and white matter hyperintensity volume was significant in *APOE4* carriers (*B* = −0.55, 95 % CI [−0.87, −0.23], *p* = .002, adjusted *p* = .003; *N* = 33; Supplemental Table III) but not non-carriers (*B* = 0.07, 95 % CI [−0.13, 0.28], *p* = .484, adjusted *p* = .484; *N* = 63; Supplemental Table IV; Fig. 2). In univariable regression, the slope for *APOE4* carriers differed significantly than the slope for non-carriers (F(1, 92) = 6.04, *p* = .016; Fig. 2).

**Table 3:** Association between EPC colony count and white matter hyperintensity volume adjusting for age and sex.

Variable	Unstandardized Coefficients		t	Sig.	95 % Confidence Interval for B	
	B	Std. Error			Lower Bound	Upper Bound
EPC Colony Count (Colonies/Well)*	−0.06	0.086	−0.68	0.499	−0.23	0.11
Age (Years)	0.02	0.004	5.50	<0.001	0.01	0.03
Sex (Male)	−0.04	0.081	−0.54	0.590	−0.21	0.12
Intracranial Volume (mL)	.0000009	.0000002	3.91	<0.001	.0000004	.000001

Dependent Variable: White Matter Hyperintensity Volume*.

*Values were log-transformed.

## Discussion

The *APOE4* gene and cerebrovascular dysfunction both increase risk for cognitive decline and dementia [16]. Emerging research suggests that endothelial dysfunction may play a key role in breakdown of the cerebral microvasculature and development of cerebral small vessel damage [16]. Fewer studies have evaluated the role of EPCs, which mobilize in response to vascular injury and may benefit the cerebrovasculature. In this study, we examined whether EPC proliferative capacity in vitro may be related to cerebral small vessel disease and white matter hyperintensity volume. Using a cell culture approach, we observed no association between EPC colony count and cerebrovascular dysfunction overall. However, in stratified analyses, EPC colony count was significantly associated with cerebral small vessel disease score and white matter hyperintensity volume, specifically in *APOE4* carriers.

These findings could suggest that *APOE4*-associated risk for cerebrovascular disease may be modified by EPC functional capacity. One previous study observed lower EPC colony counts in patients with cerebral small vessel disease [32], however no prior study has specifically examined the role of *APOE4*, which may also be a risk factor for developing cerebral small vessel disease. Fewer colonies in cell culture may suggest diminished capacity for vessel repair and greater risk for damage to the cerebrovasculature in *APOE4* carriers. Cerebral amyloid angiopathy and/or arteriosclerosis may contribute to cerebral small vessel disease in *APOE4* carriers. Alternatively, endothelial dysfunction may contribute to the development of cerebral amyloidosis and Alzheimer's disease in *APOE4* carriers vulnerable to vascular amyloid clearance and blood-brain barrier disruption [8,[36], [37], [38], [39], [40], [41], [42], [43], [44], [45]].

In vascular brain injury, the degree to which EPCs are mobilized is predictive of better neurologic outcome, and depletion of EPC reserve is associated with worse outcomes [46,47]. This is consistent with the recent Framingham Heart Study report showing lower EPC levels predict future symptoms [48]. However, the current heterogeneity in methods to quantify and define EPCs and inclusion of participants with varying levels of Alzheimer's disease severity may have contributed to the conflicting literature on the role of EPCs in Alzheimer's disease. Given that *APOE4* gene is known to impact endothelial cell function [49], future longitudinal studies of EPC markers in older adults with genetic risk for Alzheimer's disease may clarify the clinical value of EPC assays in identifying those at risk of clinical decline.

Although the clinical implications of EPC levels remain unclear, it has been hypothesized that these cells may also hold therapeutic potential for neurodegenerative conditions [[50], [51], [52]]. In experimental models of Alzheimer's disease, transplantation of in vitro cultured EPCs into APP/PS1 transgenic mice can repair the blood-brain barrier, trigger angiogenesis and decrease Aβ deposition [53,54]. However, it remains unclear whether the observed increases in EPC levels in Alzheimer's disease are beneficial, detrimental or of no impact on the disease. Given the strong association between EPCs and cerebrovascular function in *APOE4* carriers, further experimental studies should be conducted to clarify any potentially disease-modifying role for EPCs in those at risk of Alzheimer's disease.

One limitation of this study is the cross-sectional design, which limits examination of directionality or causality. Future longitudinal studies could elucidate whether elevation of EPCs and improved cerebrovascular function may limit cognitive decline in those at genetic risk of Alzheimer's disease. Moreover, we only observed a statistically significant difference in the association with EPC colony count in *APOE4* carriers versus non-carriers for white matter hyperintensity volume. However, it is possible that the analyses examining the association between EPC colony count and cerebral small vessel disease score were underpowered, given the smaller sample size for this analysis. Therefore, additional future studies with larger sampler sizes are warranted to further explore these associations in *APOE4* carriers versus non-carriers. Another possibility is that the association between EPC colony count and small vessel disease in *APOE4* carriers varies for different pathologies. Although perivascular spaces, microbleeds, lacunes and white matter lesions are all features of small vessel disease, it is possible that each has a different underlying pathophysiological mechanism and potentially a different association with EPC colony count and *APOE4* carrier status. Future studies could also examine the specific association between EPC colony count and distinct small vessel disease features in *APOE4* carriers versus non-carriers. Finally, few participants in this study had severe small vessel disease, limiting these findings to those with mild to moderate microvascular damage. Moreover, the number of *APOE4* carriers in our sample was small, which decreases statistical power and warrants future studies with larger cohorts to validate these findings.

Together, these findings suggest that *APOE4* carriers exhibit an association between EPC colony count and cerebrovascular function. These changes may ultimately increase or decrease risk of cognitive impairment and Alzheimer's disease. Future longitudinal studies are warranted to further examine whether these early changes in *APOE4* carriers are associated with development of Alzheimer's disease. Moreover, this study did not examine blood-brain barrier breakdown, which is known to contribute to the pathophysiology of cerebrovascular disease and Alzheimer's disease. Future studies are needed to further examine the association between EPCs and blood-brain barrier breakdown. The mechanism by which EPCs are associated with cerebrovascular function also remains unclear; delineating the relationship between EPCs and cerebrovascular function further may reveal the role of these factors in influencing risk of Alzheimer's disease in individuals at genetic risk of developing the disease.

## CRediT authorship contribution statement

**Arunima Kapoor:** Writing – review & editing, Writing – original draft, Methodology, Investigation, Formal analysis, Data curation. **Shubir Dutt:** Writing – review & editing, Methodology, Data curation. **Amy Nguyen:** Writing – review & editing, Methodology, Data curation. **Trevor Lohman:** Writing – review & editing, Methodology, Data curation. **Aimée Gaubert:** Writing – review & editing, Project administration, Methodology, Data curation. **John Paul M. Alitin:** Writing – review & editing, Methodology, Data curation. **Isabel J Sible:** Writing – review & editing, Methodology, Data curation. **Anisa Marshall:** Writing – review & editing, Methodology, Data curation. **Fatemah Shenasa:** Writing – review & editing, Methodology, Data curation. **Allison C Engstrom:** Writing – review & editing, Methodology, Data curation. **David Robert Bradford:** Writing – review & editing, Methodology, Data curation. **Kathleen Rodgers:** Writing – review & editing, Methodology, Data curation. **Daniel A Nation:** Writing – review & editing, Writing – original draft, Supervision, Resources, Methodology, Investigation, Funding acquisition, Conceptualization.

## Declaration of competing interest

None

## Appendix Supplementary materials

Image, application 1
