# Supplementary material for: Endothelial progenitor cells and cerebral small vessel disease in APOE4 carriers
Source: Cereb Circ Cogn Behav. 2025 Feb 11;8:100378. doi: 10.1016/j.cccb.2025.100378 (PMC11872604; doi:10.1016/j.cccb.2025.100378)
Supplement: Supplementary file 1 [file mmc1.docx]

**Supplemental Tables**

**Supplemental Table I. Association between EPC colony count and small vessel disease burden adjusting for age and sex in *APOE4* carriers**

| Variable | Unstandardized Coefficients | | t | Sig. | 95% Confidence Interval for B | |
| --- | --- | --- | --- | --- | --- | --- |
|  | B | Std. Error |  |  | Lower Bound | Upper Bound |
| EPC Colony Count (Colonies/Well)* | -1.35 | 0.57 | -2.37 | 0.026 | -2.53 | -0.18 |
| Age (Years) | 0.004 | 0.032 | 0.13 | 0.897 | -0.062 | 0.071 |
| Sex (Male) | -0.25 | 0.41 | -0.60 | 0.555 | -1.10 | 0.60 |

*Dependent Variable: Small Vessel Disease Score*

**Values were log-transformed*

**Supplemental Table II. Association between EPC colony count and small vessel disease burden adjusting for age and sex in *APOE4* non-carriers**

| Variable | Unstandardized Coefficients | | t | Sig. | 95% Confidence Interval for B | |
| --- | --- | --- | --- | --- | --- | --- |
|  | B | Std. Error |  |  | Lower Bound | Upper Bound |
| EPC Colony Count (Colonies/Well)* | -0.30 | 0.35 | -0.85 | 0.398 | -0.99 | 0.40 |
| Age (Years) | 0.04 | 0.02 | 2.75 | 0.009 | 0.01 | 0.07 |
| Sex (Male) | 0.30 | 0.28 | 1.06 | 0.295 | -0.27 | 0.87 |

*Dependent Variable: Small Vessel Disease Score*

**Values were log-transformed*

**Supplemental Table III. Association between EPC colony count and white matter hyperintensity volume adjusting for age and sex in *APOE4* carriers**

| Variable | Unstandardized Coefficients | | t | Sig. | 95% Confidence Interval for B | |
| --- | --- | --- | --- | --- | --- | --- |
|  | B | Std. Error |  |  | Lower Bound | Upper Bound |
| EPC Colony Count (Colonies/Well)* | -0.55 | 0.16 | -3.51 | 0.002 | -0.87 | -0.23 |
| Age (Years) | 0.01 | 0.01 | 0.71 | 0.486 | -0.01 | 0.02 |
| Sex (Male) | -0.27 | 0.13 | -2.14 | 0.042 | -0.53 | -0.01 |
| Intracranial Volume (mL) | .000001 | .0000003 | 3.46 | 0.002 | .0000005 | .000002 |

*Dependent Variable: White Matter Hyperintensity Volume**

**Values were log-transformed*

**Supplemental Table IV. Association between EPC colony count and white matter hyperintensity volume adjusting for age and sex in APOE4 non-carriers**

| Variable | Unstandardized Coefficients | | t | Sig. | 95% Confidence Interval for B | |
| --- | --- | --- | --- | --- | --- | --- |
|  | B | Std. Error |  |  | Lower Bound | Upper Bound |
| EPC Colony Count (Colonies/Well)* | 0.07 | 0.10 | 0.71 | 0.484 | -0.13 | 0.28 |
| Age (Years) | 0.03 | 0.01 | 5.44 | 0.000 | 0.02 | 0.04 |
| Sex (Male) | -0.06 | 0.11 | -0.54 | 0.593 | -0.27 | 0.16 |
| Intracranial Volume (mL) | .0000009 | .0000003 | 3.27 | 0.002 | .0000003 | .000001 |

*Dependent Variable: White Matter Hyperintensity Volume**

**Values were log-transformed*
